# Treatment of Moyamoya Syndrome Associated with Systemic Lupus Erythematosus and Hypothyroidism in an Adult by Encephaloduroarteriosynangiosis: A Case Report

**DOI:** 10.1155/2012/120867

**Published:** 2012-08-26

**Authors:** Arata Tomiyama, Hitoshi Kimura, Haruo Nakayama, Hideaki Izukura, Jun-ichi Harashina, Keisuke Ito, Ken-ichiro Sato, Morito Hayashi, Norihiko Saito, Takatoshi Sakurai, Yoko Hirata, Kazuya Aoki, Satoshi Iwabuchi

**Affiliations:** 2nd Department of Neurosurgery, Toho University Ohashi Medical Center, 2-17-6 Ohashi, Meguro-ku, Tokyo 104-0045, Japan

## Abstract

A 54-year-old woman presented to our hospital with progressive motor weakness of the right arm. She had a medical history of systemic lupus erythematosus (SLE) and hypothyroidism. Magnetic resonance imaging indicated a watershed infarction of the left hemisphere. Cervical echogram indicated severe stenosis of the internal carotid artery (ICA) without wall thickening. Cerebral angiography indicated left ICA occlusion, development of unilateral moyamoya vessels, and leptomeningeal anastomosis. Encephaloduroarteriosynangiosis (EDAS) was performed after cerebral _99_^m^Technetium-ethyl-cysteinate-dimer single-photon emission computed tomography indicated a decreased cerebral blood flow, diminished cerebrovascular perfusion reserve. Motor weakness finally disappeared 6 months after surgery. Moyamoya syndrome is a rare complication of both SLE and hypothyroidism, and the surgical indication remains controversial. By evaluating the decreased cerebral perfusion reserve capacity and the existence of leptomeningeal anastomosis, EDAS could be an efficient method for the treatment of moyamoya syndrome associated with SLE and hypothyroidism.

## 1. Introduction

Moyamoya-like angiopathy (also called moyamoya syndrome) is a rare intracranial complication of systemic lupus erythematosus (SLE) and hypothyroidism. However, the treatment strategy for moyamoya syndrome complicated with such chronic systemic vascular disease remains controversial. In this paper, we present a case of moyamoya syndrome associated with SLE and hypothyroidism with onset of cerebral ischemia, which was effectively treated by encephaloduroarteriosynangiosis (EDAS).

## 2. Case Presentation

 A 54-year-old woman, who had undergone treatment for SLE and hypothyroidism for 20 years by continuous steroid administration (prednisolone 5 mg/day), experienced repeating cataplexy of the right arm for 6 months and visited neurologists at our hospital. She was diagnosed with occlusion of the left internal carotid arteries (ICA) and cerebral ischemia of the left watershed area between the frontal and temporal lobe, and treatment with antiplatelets (acetylsalicylic acid 100 mg/day and cilostazol 100 mg/day) was initiated. The symptoms transiently improved, but recurred soon after. An increased dose of antiplatelets was administered (acetylsalicylic acid 200 mg/day and cilostazol 200 mg/day), but no improvement in her symptoms was observed. Subsequently, she was admitted to the department of Neurosurgery for intracranial revascularization surgery.

**Figure 1:**
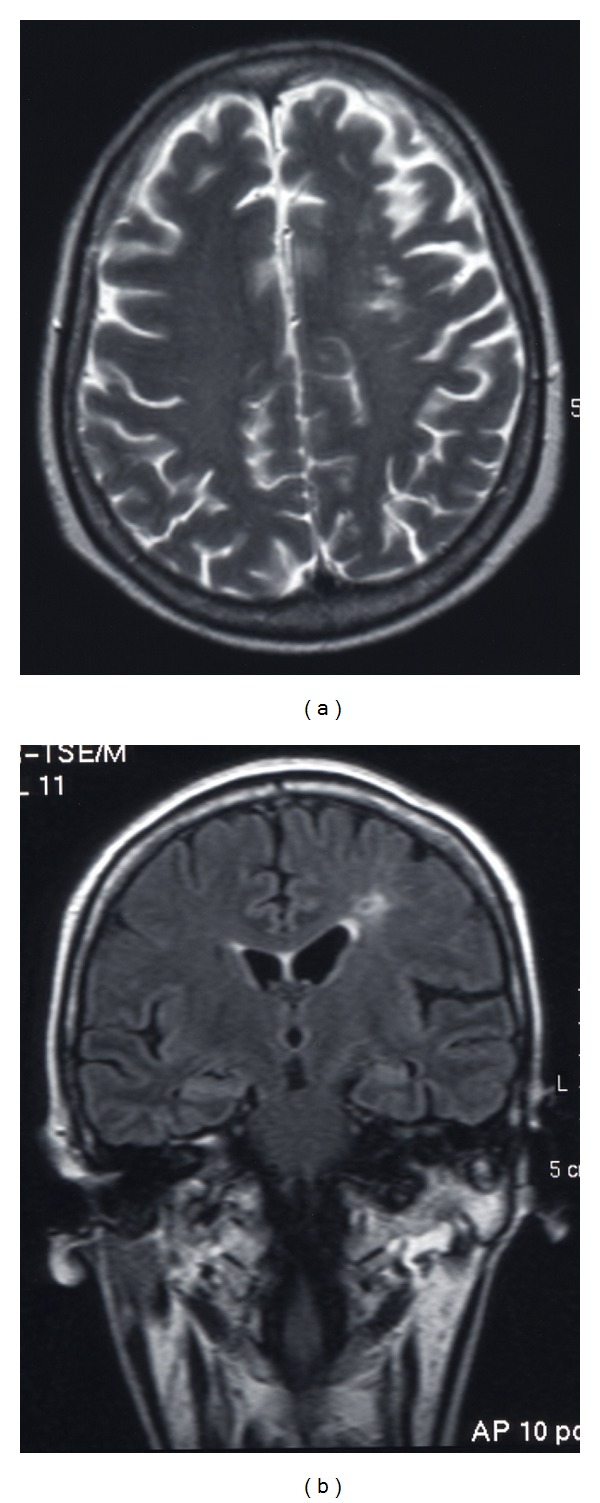
Preoperative magnetic resonance imaging axial T2-weighted image (a) and coronal FLAIR image (b). Cerebral infarction of the left fronto-parietal watershed area was noted.

**Figure 2:**
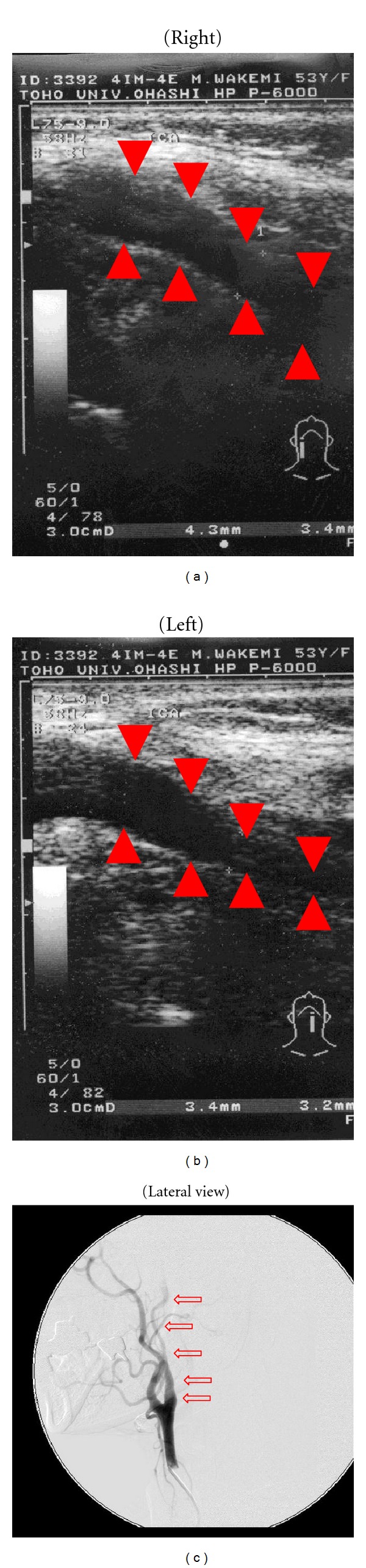
Preoperative cervical echograms (a, b) and left cervical angiogram (c). The lumen of the internal carotid artery (ICA) in echograms is indicated by arrowheads, and the left ICA in angiogram is indicated by arrows. The left cervical common carotid artery (CCA)-ICA echogram indicated severe stenosis towards the distal end of the left ICA without wall thickening (interna-media thickness = 0.8) (b) compared with the right CCA-ICA echogram (a). The lateral view of the left cervical angiography also indicated stenosis and winding of left ICA towards the distal intracranial region (c).

**Figure 3:**
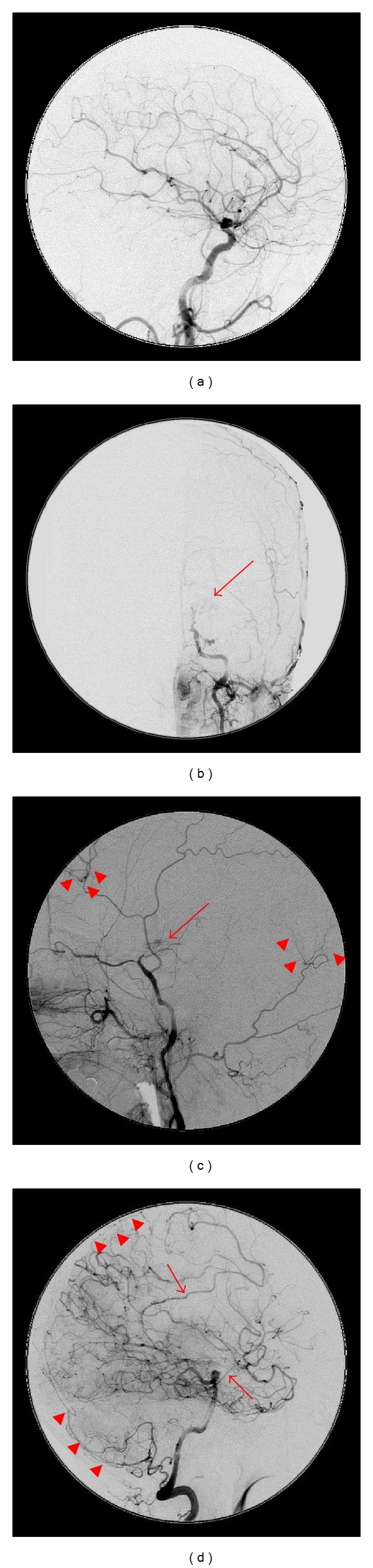
Preoperative head angiograms. Anteroposterior view (b) and lateral view of the left common carotid angiogram (c) indicating occlusion of the left internal carotid artery (ICA) at the C1 segment, leptomeningeal (transdural) anastomosis from the left external carotid artery to the left anterior cerebral artery (ACA) and middle cerebral artery (MCA) area (arrowheads), and formation of basal moyamoya vessels (arrows), and the lateral view of the right carotid angiogram (a) indicating no abnormality. Lateral view of left vertebral angiography (d) showing leptomeningeal anastomosis (arrow heads) and anastomosis to the ACA and MCA area via the left posterior cerebral artery (arrows).

**Figure 4:**
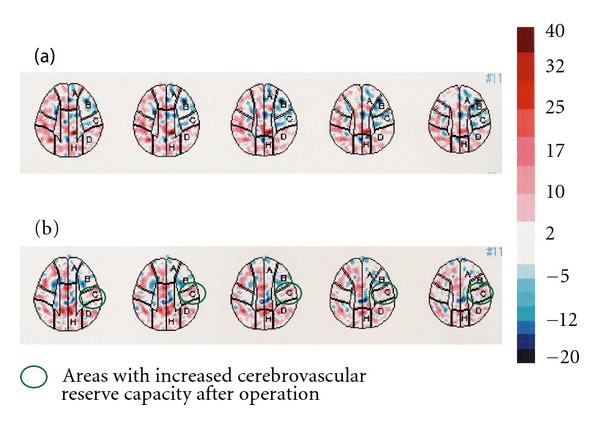
Evaluation of the increase rate of cerebral blood flow (CBF) under diamox stress using a three-dimensional stereotactic region of interest template analysis of ^99m^Technetium-ethyl-cysteinate-dimer CBF single-photon emission computed tomography (preoperative (a); postoperative (b)). Preoperative CBF increase rate under diamox stress was impaired more around the central sulcus than on the ipsilateral side (2.2% and 10.8%, resp.). Postoperative CBF increase rate by acetazolamide stress around the left central sulcus was improved (5.7%, green circle) compared with that observed during the preoperative study.

Preoperative head magnetic resonance imaging indicated cerebral infarction of the frontoparietal watershed area (Figures 1(a) and 1(b)). Carotid echogram indicated shrinkage of the left ICA towards the distal end, starting at the level of carotid bifurcation without vascular wall thickening (Figure 2(b)), compared with the right ICA (Figure 2(a)); the possibility of steroid-induced atherosclerosis as the cause of this left ICA occlusion was dismissed. And, left cervical digital subtraction angiography (DSA) also indicated stenosis and winding of the left cervical ICA towards the distal intracranial lesion (Figure 2(c)). Head DSA of the right ICA indicated no abnormality (Figure 3(a)). However, DSA of the left common carotid artery indicated the occlusion of the left ICA just after the bifurcation of the posterior communicating artery, deficit of the left anterior cerebral artery and the left middle cerebral artery, development of moyamoya-like vessels in the left basal ganglia (Figures 3(b) and 3(c)), and leptomeningeal anastomosis from the left posterior cerebral artery to the perfusion area of the left anterior and middle cerebral artery (Figure 3(d)). Analysis of ^99m^Technetium-ethyl-cysteinate-dimer single-photon emission computed tomography (^99m^Tc-ECD SPECT) imaging of the cerebral blood flow (CBF) using three-dimensional stereotactic region of interest template highlighted a reduced level of CBF in the left hemisphere under acetazolamide loading, compared with the right hemisphere (Figure 4(a)).

On the basis of these results, surgical intervention for cerebral ischemia was considered necessary, due to moyamoya syndrome and left ICA occlusion. EDAS—a method of revascularization surgery—is an indirect procedure and was chosen because this case was complicated by the systemic progressive vascular disease that was treated with steroids, and the preoperative status of the left middle cerebral artery, which was not visualized by DSA, was not known.

**Figure 5:**
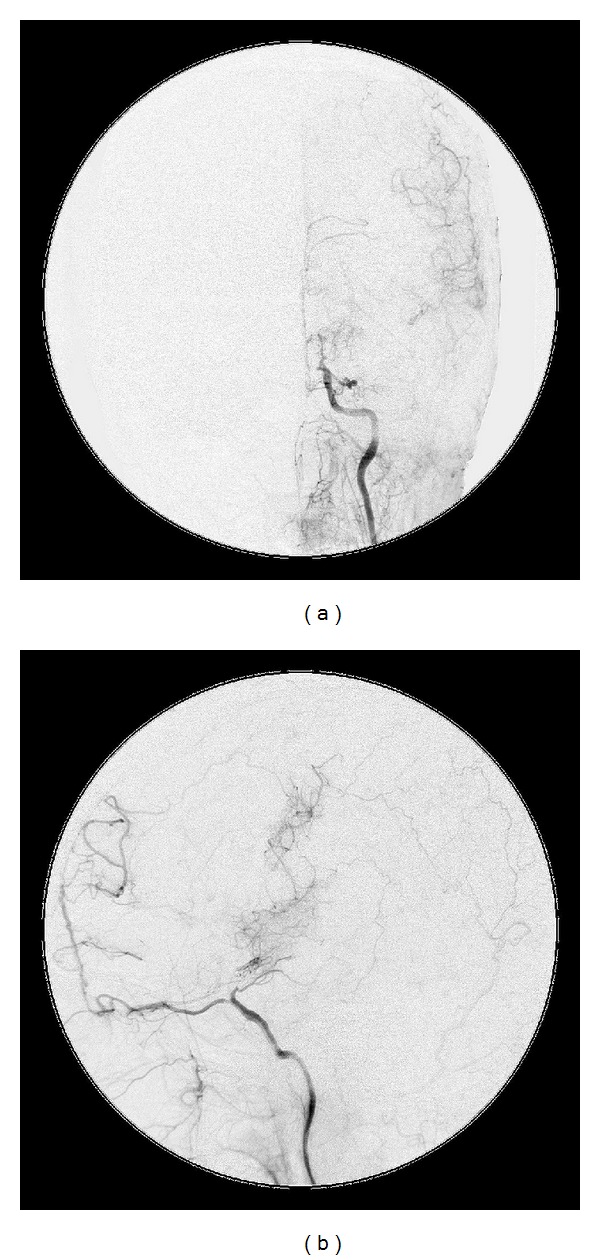
Postoperative digital subtraction angiography. Anteroposterior view (a) and lateral view (b) of the left common carotid angiogram indicating marked neovascularization from the graft compared with preoperative angiography (Figures 3(b) and 3(c)).

The postoperative course was uneventful, and the cataplexy of the right upper limb had nearly disappeared. A postoperative DSA study indicated marked neovascularization from the graft (Figures 5(a) and 5(b)). Postoperative SPECT imaging of the CBF under acetazolamide loading indicated improvement in cerebrovascular reserve capacity of the left hemisphere, particularly around the central sulcus (Figure 4(b)).

## 3. Discussion

 Intracranial moyamoya-like vessels complicated with systemic vascular disease or immunological disorders, such as in this case, are diagnosed as “moyamoya syndrome,” rather than true moyamoya disease according to the precise criteria, and the exact pathology remains unknown [1, 2]. Natori et al. named this vasculopathy as “angiographical moyamoya” and indicated the relationship between the pathogenic mechanisms of moyamoya syndrome and true moyamoya disease at the genetic level in their report [3]. Complication of SLE with moyamoya syndrome is rare, and only 3 or more cases of moyamoya syndrome complicated with SLE have been reported [4–6], which were treated by direct or indirect bypass surgery. Most disorders of large cerebral vessels associated with SLE are reported as thrombus, dissection, fibromuscular dysplasia or vasculitis, and atherosclerosis [6]. Another possible pathogenic mechanism for moyamoya syndrome complicated with SLE is progressive atherosclerosis induced by continuous steroid treatment for SLE. Given that we observed gradual stenosis of the ICA from the cervical level towards the distal end without wall thickening (Figure 2(b)), it is unlikely that the pathogenesis for our case is steroid-induced atherosclerosis. The cases of moyamoya syndrome complicated with hypothyroidism are reported as Sjögren's syndrome with cerebral moyamoya vessels [5, 7, 8]. In these cases, autoimmune disorder is suggested as the common pathogenesis between moyamoya syndrome and Sjögren's syndrome. However, despite these findings, the correlation in the pathogenesis of moyamoya syndrome and SLE or hypothyroidism remains unclear.

 The surgical strategy against moyamoya syndrome complicated with systemic vasculopathy is still controversial, particularly for adult cases. Kaga et al. reported a case of adult moyamoya syndrome with reduced cerebrovascular reserve capacity, which was effectively treated by an indirect method [9]. Moreover, Isono et al. reported 5 cases of moyamoya syndrome with reduced cerebrovascular reserve capacity, which were treated by indirect revascularization surgery; they concluded that indirect methods were effective only in cases with well-developed leptomeningeal anastomosis [10]. However, El Ramahi and Al Rayes reported a case of moyamoya syndrome complicated with SLE, which was treated by an indirect method [4]. Consequently, their case was complicated by intracerebral hemorrhage after the operation, and no remarkable therapeutic effect was achieved. However, their case did not evaluate the preoperative cerebrovascular reserve capacity. Our present case indicated not only well-developed leptomeningeal anastomosis, but also decreased cerebrovascular reserve capacity of the ischemic area through preoperative examination. Therefore, careful consideration of the preoperative angiographical findings and cerebrovascular reserve capacity would be vital for determining the indication of indirect revascularization surgery for moyamoya syndrome complicated with systemic progressive vascular disease.

## 4. Conclusion

By evaluating preoperative cerebrovascular reserve capacity and the development of leptomeningeal anastomosis, the case of moyamoya syndrome associated with progressive vasculopathy would be treated effectively by indirect revascularization surgery, including EDAS, despite the earlier continuous steroid treatment.
